# Hepatoprotective drug screening identifies daclatasvir, a promising therapeutic candidate for MASLD by targeting PLIN2

**DOI:** 10.1016/j.jlr.2025.100835

**Published:** 2025-05-29

**Authors:** Rui Shu, Song Tian, Weiyi Qu, Jinjie Yang, Wei Shi, Xinyan Li, Toujun Zou, Changjin Jiang, Yuxuan Zhang, Zifeng Yang, Han Tian, Hailong Yang, Jiajun Fu, Zhi-Gang She, Hongliang Li, Xiao-Jing Zhang

**Affiliations:** 1 Wuhan University TaiKang Medical School (School of Basic Medical Sciences), Wuhan, China; 2 State Key Laboratory of New Targets Discovery and Drug Development for Major Diseases, Gannan Innovation and Translational Medicine Research Institute, Ganzhou, China; 3 School of Pharmacy, Gannan Medical University, Ganzhou, China; 4 Department of Cardiology, Zhongnan Hospital of Wuhan University, Wuhan, China; 5 Qujing Medical College Basic Medical Department, Qujing, China; 6 Department of Cardiology, Renmin Hospital of Wuhan University, Wuhan, China

**Keywords:** MASH, daclatasvir, PLIN2, ubiquitination, protein degradation

## Abstract

Metabolic dysfunction-associated steatohepatitis (MASH) has become a global health challenge with limited therapeutic strategy. Here, this study aims to identify promising drug candidates for MASH and clarify its pharmacological mechanism. By extensive screening of FDA-approved hepatoprotective medicines using a PA/OA-stimulated hepatocytes model, we identified daclatasvir showing potent anti-MASH capacity against hepatic steatosis deposition and inflammatory response. The hepatoprotective benefits of daclatasvir were further validated in MASH mouse models, induced by a high-fat high-cholesterol (HFHC) diet for 16 weeks or a methionine-choline-deficient (MCD) diet for 4 weeks, as supported by markedly improved histopathological characteristics, serum biochemical level, and transcriptomic analyses. Using the molecular docking assay followed by isothermal titration calorimetry confirmation, we identified that daclatasvir functions as a new perilipin-2 (PLIN2) inhibitor by interrupting its stability. In specific, PLIN2 subjected to MARCH6-mediated protein degradation in a K11-type ubiquitination. Daclatasvir can directly bind to PLIN2 and enhance its interaction with MARCH6, leading to markedly strengthened PLIN2 ubiquitinational degradation and the subsequent decline in lipid droplet disintegration and lipotoxicity. The specific mutation at the binding amino acid sites of PLIN2 with daclatasvir largely abolished the anti-MASH benefit of daclatasvir. In conclusion, the findings of our study for the first time identified the anti-HCV drug daclatasvir as a novel and potent PLIN2 protein degradant for protection against MASH.

Metabolic-dysfunction associated steatotic liver disease (MASLD) is the most prevalent chronic liver disease, affecting approximately 32.4% of the global population and showing a rapid increase in prevalence (1, 2). Beyond liver-specific complications, MASLD and its advanced form metabolic dysfunction-associated steatohepatitis (MASH) is closely associated with extrahepatic disorders, including cardiovascular disease, type 2 diabetes, chronic kidney disease and cancer, making it a heavy health burden in public (3, 4). While lifestyle interventions are effective in early-stage MASLD, their efficacy largely diminishes in advanced cases (5, 6). Hitherto, only resmetirom has been approved for MASH therapy that should be accompanied by exercise and dietary change, with a resolving rate limited to less than 30% (7). The insufficient therapeutic strategies underscore an urgent need for therapeutic drug candidates for MASH.

The new drug development is time-consuming and requires huge financial resources, with a relatively low success rate. As an alternative, drug repurposing provides a more efficient strategy to reduce development time and costs by identifying new therapeutic applications for existing drugs (8, 9). This approach takes advantage of the established pharmacodynamic, pharmacokinetic, and toxicological profiles of approved drugs, allowing for the rapid identification of treatments for unmet clinical needs (10). For instance, the anti-diabetic sodium-glucose cotransporter 2 (SGLT2) inhibitors have shown unexpected benefits in treating heart failure (11) and that statins, originally used for hyperlipidemia, exhibit anti-inflammatory and antifibrotic effects in conditions such as chronic kidney disease (12). In our preliminary studies, to identify potential anti-MASH drug candidates, we have screened the FDA-approved drug library and identified daclatasvir, the antiviral medication used for chronic hepatitis C infection, showed robust inhibitory effect on MASH progression.

Daclatasvir (DCV) is the first clinically validated HCV non-structural protein 5A (NS5A) inhibitor that blocks viral replication by preventing genome transfer to assembly sites and inhibiting replication complex formation (13). It has stable pharmacokinetics, unaffected by factors such as age, gender, hepatic or renal function, food intake, or pH, making it highly versatile for clinical use (14). In addition, daclatasvir exhibits minimal interactions with commonly prescribed drugs, enabling safe and effective combination with other antiviral agents to improve treatment outcomes for HCV patients (15). Beyond its well-established antiviral activity, daclatasvir combined with sofosbuvir has been shown to exert significant antifibrotic effects, independent of its role in viral inhibition. These effects include reductions in fibrosis markers, collagen deposition, and hepatic stellate cell activation, underscoring its potential for broader therapeutic benefits (16). Notably, anti-HCV drugs possess potential benefits in regulating lipid homeostasis and insulin signaling (17, 18). However, the effects of daclatasvir on MASLD-related lipid and metabolic disturbances remain far from fully clarified.

In the present study, we clearly demonstrated the anti-MASH capacity of an anti-HCV medicine daclatasvir by directly targeting lipid droplet stability. The detailed mechanisms of daclatasvir on MASH have been revealed as promoting MARCH6-mediated PLIN2 ubiquitination and degradation. These findings underscore the potential of repurposing existing therapies of daclatasvir for metabolic liver diseases and open new avenues for addressing the unmet clinical needs of patients with MASLD and MASH.

## Materials and Methods

### Animals and treatment

The C57BL/6J mice used in this study were purchased from Vital River and housed in an SPF (Specific Pathogen Free) environment, with unrestricted access to food and water. The environmental conditions were maintained at a constant temperature of 23°C ± 2°C, with humidity levels between 55% and 65% and a 12-h light/dark cycle. Animal experiments were reviewed and approved by the Animal Care and Use Committee of Renmin Hospital of Wuhan University (permission number: 20221004C). Personnel conducting the experiments were fully qualified.

In this study, two diet-induced mouse models were employed to assess the therapeutic effects of daclatasvir on the progression of MASH. One was established by feeding mice a high-fat, high-cholesterol (HFHC) diet for 16 weeks, and the other was established by feeding a methionine-choline-deficient (MCD) diet for 4 weeks, with mice fed a normal chow (NC) diet as the control group. For daclatasvir treatment, the drug was administered intragastrically to the mice at a dose of 10 mg/kg body weight per day in 1% sodium carboxymethylcellulose. The 10 mg/kg dose was chosen based on preclinical studies showing its efficacy in liver disease models and its ability to achieve therapeutic plasma concentrations with a safe margin (19, 20, 21). The vehicle control was given a blank solution. Before sampling, the mice were euthanized by cervical dislocation after anesthesia with 5% pentobarbital.

### Histopathological analysis

Tissue samples were collected from the liver, fixed in 10% formalin at room temperature for 48 h, and then dehydrated and embedded in paraffin, or directly embedded in an optimal cutting temperature compound (OCT, 4583; Sakura). Hematoxylin and eosin (H&E) staining (C0105M; Beyotime) was used to assess tissue morphology and overall structure. Lipid deposition detection and measurement by Oil red O (O0625; Sigma-Aldrich) staining of OCT-embedded liver sections. Liver fibrosis was assessed using Picrosirius Red staining (26357-02; HeDe Biotechnology). The abovementioned operations were conducted under an optical microscope (Olympus), and corresponding histopathological images were captured. Immunofluorescence staining was performed on paraffin-embedded tissue sections. For paraffin sections, antigen retrieval was carried out using EDTA-based heat-induced epitope retrieval. Sections were then blocked with 8% goat serum for 1 h at room temperature to reduce nonspecific staining. The sections were incubated with a rabbit anti-CD11b primary antibody (BM3925, Boster) overnight at 4°C, followed by incubation with a goat anti-rabbit fluorophore-conjugated secondary antibody (A-11036, Invitrogen). Images were captured using a Nikon fluorescence microscope (ECLIPSE 80i), and the percentage of CD11b-positive cells was quantified using ImageJ software.

### Serum biochemical analysis

To assess lipid levels and liver function in mice, serum concentrations of triglycerides (TG), total cholesterol (TC), alanine aminotransferase (ALT), and aspartate aminotransferase (AST) were measured. All analyses were performed using the ADVIA 2400 Chemistry System analyzer (Siemens) following the manufacturer’s instructions.

### Cell culture

The HuH-7 and 293T cell lines used in this study were obtained from the Cell Bank of the Chinese Academy of Sciences. The cells were cultured in Dulbecco's Modified Eagle Medium (DMEM; C11995500BT; Gibco) supplemented with 10% fetal bovine serum (FBS;10099-141; Gibco), 100 μg/ml penicillin, and 100 μg/ml streptomycin (15,140-122; Gibco). The cells were incubated at 37°C in a humidified atmosphere with 5% CO_2_. Cells were used between passages 5 and 20 to maintain cellular characteristics and ensure reproducibility of results.

### Plasmid construction

Plasmids encoding full-length human *PLIN2*, *MARCH6*, and *UBR1* were constructed by cloning the respective cDNA sequences from NCBI (supplemental Table S1) into the pHAGE vector using the respective primers. The human *PLIN2* point mutation (Ser229, Glu241) plasmid was generated using mutation-specific primers for inverse PCR, while the sh*PLIN2* knockdown plasmid was constructed by cloning sh*PLIN2*-specific primers into the pLKO.1 vector. All constructs were confirmed by DNA sequencing. The primers used for plasmid construction are detailed in supplemental Table S2.

### Construction of cell lines

The sh*PLIN2* plasmid, along with the packaging plasmids pMD2.G and psPAX2, was co-transfected into 293T cells. Forty-eight hours post-transfection, the lentiviral supernatant was harvested and filtered. Target cells were seeded to 60% confluence the day before infection. The viral supernatant supplemented with 10 μg/ml polybrene was added to the target cells. After 18 h of incubation, the medium was replaced. Forty-eight hours post-infection, the target cells were transferred into new dishes containing 2 μg/ml puromycin (ST551; Beyotime) to select knockdown cells. The efficiency of the knockdown was evaluated using Western blot analysis.

### Nile Red staining of cells

Cells (10,000–20,000 cells per well) were seeded into 24-well plates with coverslips. After cell adhesion, 10 μM daclatasvir or an alternative FDA-approved drug from the library (#L1300, Selleckchem) was added to the culture medium, along with an equivalent volume of DMSO (D2650; Sigma) as a vehicle control. The cultures were pre-incubated for 6 h to ensure uniform drug exposure, followed by the addition of complete medium containing 0.25 mM PA (P0500; Sigma), 0.5 mM OA (O1383; Sigma), and 0.5% BSA (BAH66-0100; Equitech Bio). For the post-incubation treatment, cells were first stimulated with PA/OA for 6 h, then treated with DCV for an additional 8–12 h before subsequent experiments. The control group received complete medium with 0.5% BSA only. After 18 h of PA/OA stimulation, the medium was discarded, and cells were washed twice with phosphate-buffered saline (PBS). Cells were fixed with 4% paraformaldehyde (BLRE150, Biolight) for 30 min a room temperature and washed twice with PBS. Nile red stock solution (19123; Sigma) was diluted to 1 μM with PBS, and the working solution was added to each well. After 10 min at room temperature, the cells were washed twice with PBS. Coverslips were removed and mounted onto slides with DAPI (S36939; Invitrogen). Cells were observed and photographed using a confocal fluorescence microscope. All procedures were conducted under light-free conditions.

### Cell viability assay

HuH-7 cells were seeded (2000 cells per well) in a 96-well plate. Upon reaching approximately 70% confluence, the cells were treated with varying concentrations of daclatasvir (0 μM, 2.5 μM, 5 μM, 10 μM, 20 μM, and 40 μM). After 24 h of treatment, cell counting was performed using Cell Counting Kit-8 (B34304; Bimake). The absorbance at 450 nm was measured to determine the cell viability.

### Isolation and treatment of primary mouse hepatocytes

Primary hepatocytes were isolated from C57BL/6J mice using a two-step collagenase perfusion method. The liver was perfused via the portal vein with calcium-free HBSS, followed by a solution containing collagenase for enzymatic digestion. After digestion, the liver was excised, gently dissociated, filtered through a cell strainer, and centrifuged to collect hepatocytes. Cell viability was assessed using trypan blue exclusion and typically exceeded 85%. The isolated hepatocytes were then seeded in Dulbecco’s Modified Eagle Medium (DMEM; C11995500BT; Gibco) supplemented with 10% fetal bovine serum (FBS; 10099-141; Gibco), 100 μg/ml penicillin, and 100 μg/ml streptomycin (15140-122; Gibco), and cultured at 37°C in a humidified atmosphere of 5% CO_2_ for 24 h to allow cell attachment. To induce lipid accumulation, cells were switched to serum-free DMEM and treated with 0.5 mM palmitic acid (PA; P0500, Sigma) conjugated to BSA for 8–12 h.

### Detection of intracellular TG and TC

Cells are lysed in 0.1 mol/L PBS (pH 7.0–7.4) to release TG and TC. These are quantified using commercial assay kits (A110-1-1 and A111-1-1, Nanjing Jiancheng) containing enzymes that convert TG and TC into hydrogen peroxide, which forms a colored complex with 4-aminoantipyrine and hydroquinone. Absorbance is measured at 500 nm using a SpectraMax microplate reader, and concentrations are calculated based on standard curves from the kits.

### RNA extraction and real-time PCR

Total RNA was extracted from liver cells or tissues using TRIzol reagent (T9424; Sigma-Aldrich). After extraction, RNA purity and concentration were measured with a NanoDrop 2000 spectrophotometer (840–317400; Thermo Scientific). Following this, 2 μg of RNA was reverse-transcribed into cDNA using the PrimeScript RT reagent kit (R101-01; Vazyme). Subsequently, quantitative PCR was performed with SYBR Green PCR Master Mix (Q121-02; Vazyme) on a LightCycler 480 real-time PCR system (Roche). Each sample was measured in triplicate to ensure accuracy. The relative expression levels of the target genes were then normalized with reference to *ACTB* (for human genes) or Actb (for mouse genes), which was selected as the most stably expressed reference gene after comparison with other commonly used housekeeping genes. The primers used for quantitative RT-PCR (qRT-PCR) are listed in supplemental Table S3.

### RNA-sequencing and data analysis

Total RNA was extracted from samples using TRIzol reagent, and RNA quality was assessed with the Agilent RNA 6000 Nano Kit (5067-1511, Agilent Technologies). Library preparation was performed using the MGIEasy RNA Library Preparation Kit (1000006384, MGI Tech Co.). Clean reads were aligned to the mouse reference genome (GRCm38/mm10) using HISAT2 (v2.1.0) and sorted into BAM format with SAMtools. Gene expression levels were calculated as FPKM values using StringTie (v1.3.3). Differentially expressed genes (DEGs) were identified with DESeq2 (v1.32.0), considering those with adjusted *P*-values <0.05 and log2(fold change) ≥ log2(1.5). KEGG pathway enrichment analysis for DEGs was conducted using Perl scripts.

### Western blotting

Cells or tissue samples were washed with ice-cold PBS buffer and lysed with RIPA lysis buffer (65 mM Tris-HCl, pH 7.5; 150 mM NaCl; 1 mM EDTA, pH 8.0; 1% NP-40, 0.5% sodium deoxycholate, and 0.1% SDS) containing protease inhibitors tablets (04693132001, Roche) and phosphatase inhibitor tablets (4906837001; Roche). Protein concentration was measured using a BCA protein assay kit (23225, Thermo Fisher Scientific). Proteins were separated by electrophoresis using a 10% SDS-PAGE gel, transferred to a PVDF membrane (IPVH00010, Millipore), and blocked with 5% non-fat milk for 1 h. The membrane was incubated with the primary antibody overnight at 4°C, followed by incubation with an HRP-conjugated secondary antibody at room temperature for 1 h. Imaging was performed using a ChemiDoc MP Imaging System (Bio-Rad; Hercules). The antibodies used are detailed in supplemental Table S4.

### Immunoprecipitation assays

293T cells were transfected with the indicated plasmids before lysis with IP lysis buffer (20 mM Tris-HCl, pH 7.4; 150 mM NaCl; 1 mM EDTA, pH 8.0%; and 1% NP-40) containing protease inhibitor and phosphatase inhibitor tablets. For each IP sample, 500 μl of lysate was incubated overnight at 4°C with 10 μl of protein A/G agarose beads (11719394001, 11719386001; Roche) and 1 mg of the indicated antibody on a rocking platform. The beads were washed at least three times with cold NaCl buffer and boiled with 2×SDS loading buffer before analysis by Western blotting.

### Ubiquitination assay

HEK 293T cells were plated and grown to 60% confluence before transfection with overexpression plasmids. Change the medium 6–8 h after transfection. At 24 h post-transfection, treat the cells with drugs. When collecting samples, wash the cells with PBS, scrape them into new tubes and centrifuge. Discard the supernatant and add IP buffer containing protease inhibitor and phosphatase inhibitor. After centrifugation, the supernatant was collected. For input samples, a portion of the supernatant was mixed with an SDS loading buffer and heated. For immunoprecipitation, Protein A/G beads were incubated with an antibody, then added to the lysate and rotated at 4°C. The mixture was centrifuged and washed three times with NaCl IP buffer, and each wash was followed by centrifugation. Finally, after discarding the supernatant, the beads were mixed with SDS loading buffer, heated, and used for further analysis.

### Molecular docking

The 3D structures of target proteins were downloaded from the RCSB PDB database (https://www.rcsb.org/). Water molecules and heteroatoms were removed, while key cofactors were retained. Proteins were prepared for docking by protonation, hydrogen atom addition, and Gasteiger charge assignment using AutoDockTools. The daclatasvir structure was built with Chem3D and minimized energy. Docking was performed with AutoDock Vina (https://vina.scripps.edu/), setting grid parameters around the proteins' active or binding sites. Multiple binding conformations were generated, and binding free energies were calculated. Schrödinger Glide XP was then used to calculate virtual screening scores (XP Gscore), and Prime MM-GBSA was used to compute the binding free energy change (ΔG). Proteins with high XP Gscore and favorable MM-GBSA values were identified as potential daclatasvir targets. The top-ranking complexes with the lowest binding energies were selected for further experimental validation.

### Protein purification

The recombinant plasmid pET-28a(+)-PLIN2-His, containing the full-length human PLIN2 coding sequence with an N-terminal 6×His tag, was verified by sequencing and subsequently transformed into Escherichia coli NiCo21(DE3). The bacteria were cultured in Luria-Bertani (LB) medium containing 50 μg/ml kanamycin at 37°C until reaching an OD_600_ of 0.6–0.8, followed by induction with 0.5 mM IPTG at 16°C overnight. The expressed recombinant protein was purified to >90% purity (yield: 15–20 mg/L), as confirmed by SDS-PAGE and mass spectrometry. Cells were collected and lysed on ice using lysis buffer (50 mM Tris at pH 8.0, 150 mM NaCl, 10 mM imidazole, 1 mM MgCl_2_, PMSF protease inhibitor) by sonication. The lysate was centrifuged at 16,000 *g* for 30 min at 4°C to obtain the supernatant. His-tagged proteins were purified using a Ni-NTA column (H-350-5; Golden Biotechnology) after washing with buffer A (50 mM Tris at pH 8.0, 150 mM NaCl, 10 mM imidazole) and buffer B (50 mM Tris at pH 8.0, 150 mM NaCl, 20 mM imidazole), the bound fraction was eluted using buffer C (50 mM Tris at pH 8.0, 150 mM NaCl, 250 mM imidazole). The eluted fraction was dialyzed against PBS and further purified by ultrafiltration. Protein purity and concentration were verified by SDS-PAGE and the Bradford method. The purified protein was stored in a buffer containing 20 mM Tris-HCl at pH 8.0, 100 mM NaCl, and 10% glycerol at −80°C.

### Isothermal titration calorimetry (ITC)

Isothermal titration calorimetry was employed to investigate the binding interactions between daclatasvir and PLIN2. PLIN2 protein was dissolved in PBS containing 1% DMSO and placed into the calorimetric cell, while a daclatasvir solution, prepared in the same buffer, was loaded into the titration syringe. The total titration volume was 250 μl, with 50 μl of titrant injected at 300-s intervals. Stirring was maintained at 250 rpm, and each injection delivered 2.5 μl of the daclatasvir solution. To account for heat changes unrelated to binding, a background titration was conducted using PBS (1% DMSO) alone. Data were analyzed using Nano Analyze software, and key binding parameters, including binding affinity (Kd), enthalpy (ΔH), and stoichiometry (n), were calculated via nonlinear regression based on a single-site binding model. All experiments were performed in triplicate to ensure the reliability and reproducibility of the results.

### Statistical analysis

Statistical analyses were conducted using IBM SPSS software (version 25.0), and results are presented as mean ± SD. Normality tests were first performed for all groups using the Shapiro–Wilk test. For normally distributed data, comparisons between two groups were analyzed with a two-tailed Student's *t* test, and comparisons between multiple groups were analyzed by using a one-way analysis of variance (ANOVA) with Bonferroni post hoc test (for data showing homogeneity of variance) or Tamhane's T2 (M) post hoc test (for data showing heteroscedasticity). For skewed distributions, data were analyzed using nonparametric tests: the Mann–Whitney *U* test for two groups and the Kruskal–Wallis test for multiple groups. *P* values < 0.05 were considered significant.

## Results

### Daclatasvir was screened out as a potential therapeutic agent for MASLD

**Fig. 1:**
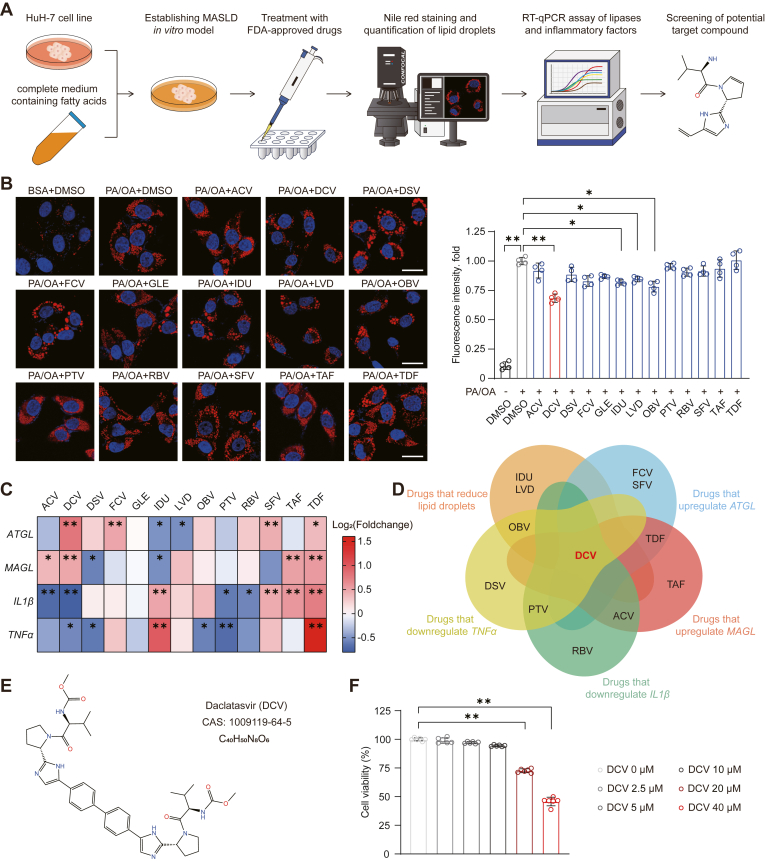
Screening strategy and functional validation of candidate drugs for MASLD treatment. A: Schematic representation of the characterization and screening strategy employed for the FDA-approved drug library. B: Representative Nile red staining images and quantitative analysis of HuH-7 cell MASLD models in the stimulation of palmitic acid/oleic acid (PA/OA, 0.25 mM/0.5 mM) and in the treatment respectively with 13 candidate drugs for 18 h. Scale bar, 25 μm. Each group was compared with the PA/OA+dimethyl sulfoxide (DMSO) group. C: Heatmap of fold changes representing the mRNA levels of *ATGL*, *MAGL*, *IL1β*, and *TNFα* in HuH-7 cell MASLD models challenged with PA/OA (0.25 mM/0.5 mM) and treated with the candidate drugs for 18 h. The PA/OA+DMSO group was served as the control. D: Venn diagram showing the overlap of drugs that reduced lipid droplets in (B), those that increased the expression of *ATGL* and *MAGL*, and decreased *IL1β* and *TNFα* expression in (C). E: Chemical structure, CAS number, and simplified structure of daclatasvir. F: Cell viability following 24 h-treatment with daclatasvir at the indicated concentrations in HuH-7 cells. Each group was compared with the DMSO group (labeled as daclatasvir 0 μM on the figure). For (B), n = 4 independent biological replicates; for (C), n = 3 independent biological replicates; for (F), n = 6 independent biological replicates. Data are presented as mean ± SD; ∗*P* < 0.05, ∗∗*P* < 0.01; one-way ANOVA in (B, C, F). BSA, bovine serum albumin; ACV, acyclovir; DCV, daclatasvir; DSV, dasabuvir; FCV, famciclovir; GLE, glecaprevir; IDU, idarubicin; LVD, lamivudine; OBV, ombitasvir; PTV, paritaprevir; RBV, ribavirin; SFV, sofosbuvir; TAF, tenofovir alafenamide; TDF, tenofovir disoproxil fumarate.

To identify potential drugs for MASLD treatment, we performed a screening and characterization of FDA-approved hepatoprotective medications using an in vitro MASLD model developed from HuH-7 human hepatocellular carcinoma cells (Fig. 1A). The following compounds were used for screening experiments, including acyclovir, daclatasvir, dasabuvir, famciclovir, glecaprevir, idarubicin, lamivudine, ombitasvir, paritaprevir, ribavirin, sofosbuvir, tenofovir alafenamide, and tenofovir disoproxil fumarate. The potential anti-MASLD function of drug candidates was first evaluated by Nile Red staining-labelled changes in intracellular lipid droplets. Notably, daclatasvir, idarubicin, lamivudine, and ombitasvir exhibited significant lipid degradation (Fig. 1B). We further examined their regulatory functions at gene expression panel that mainly reflected by transcript levels of lipases (ATGL, MAGL) and inflammatory genes (IL1β, TNFα) in drug-treated cells (Fig. 1C). Venn analysis was then employed to identify drugs showing comprehensive benefits on lipolysis, inflammation and lipid droplets degradation (Fig. 1D). Among these, daclatasvir uniquely demonstrated all three above-mentioned properties (Fig. 1E). A cell viability assay was performed to determine the available concentration range of daclatasvir for cell treatment, which indicated no significant cytotoxicity at drug concentrations ranging from 2.5 to 10 μM (Fig. 1F).

### Daclatasvir ameliorates lipid accumulation and suppresses inflammatory response in MASLD cell models

**Fig. 2:**
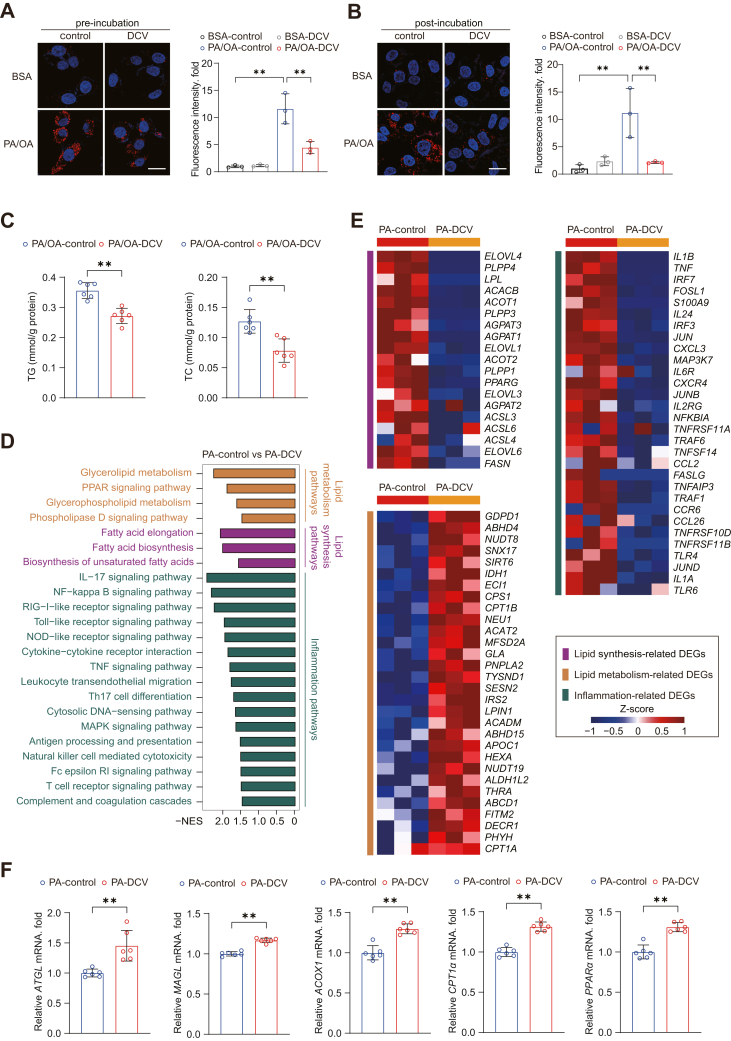
Daclatasvir mitigates lipid accumulation and inflammation in MASLD cell models. A: Representative images of Nile Red staining and quantitative analysis of lipid accumulation in HuH-7 cells pretreated with daclatasvir for 6 h, followed by co-treatment with PA/OA (0.25 mM/0.5 mM) for 18 h. Scale bar, 25 μm. B: Representative images and quantification of lipid accumulation in HuH-7 cells first stimulated with PA/OA (0.25 mM/0.5 mM) for 6 h, followed by daclatasvir treatment for an additional 8–12 h. Scale bar, 25 μm. C: Triglyceride (TG) and total cholesterol (TC) levels in HuH-7 cells treated with or without daclatasvir after 18 h of PA/OA (0.25 mM/0.5 mM) stimulation. D and E: KEGG enrichment analysis showed pathways (D) and heatmap showed the expression of differentially regulated genes (E) associated with lipid metabolism and inflammation, in HuH-7 cells challenged with PA (0.25 mM) and treated with daclatasvir for 12 h. F: Quantitative polymerase chain reaction (qPCR) assay of *ATGL*, *MAGL*, *ACOX1*, *CPT1α*, and *PPARα* in HuH-7 cells challenged with palmitic acid (PA, 0.25 mM) and treated with or without daclatasvir for 12 h. For (A, B), (D,E), n = 3 independent biological replicates; for (C, F), n = 6 independent biological replicates. Data are presented as mean ± SD; ∗*P* < 0.05, ∗∗*P* < 0.01; one-way ANOVA in (A), and Student’s *t* test in (C, F).

To further investigate the therapeutic effects of daclatasvir, we performed Nile Red staining after drug treatment on HuH-7 cells in response to PA/OA challenge. The quantitative analysis indicated that daclatasvir significantly inhibited lipid accumulation induced by fatty acid overload (Fig. 2A, B). Measurements of triglycerides (TG) and total cholesterol (TC) confirmed that daclatasvir effectively reduced content of lipids in HuH-7 cells (Fig. 2C). Additionally, transcriptomic analysis showed significant improvements in pathways related to lipid synthesis, lipid metabolism and inflammation in HuH-7 cells after daclatasvir treatment (Fig. 2D, E). Further analysis revealed that daclatasvir treatment significantly increased the transcription of key genes related to lipolysis and fatty acid β-oxidation in HuH-7 cells, including *ATGL*, *MAGL*, *ACOX1*, *CPT1α*, and *PPARα* (Fig. 2F). Thus, daclatasvir effectively improves lipid homeostasis and alleviates inflammatory responses in HuH-7 cells.

### Daclatasvir ameliorates diet-induced MASH in mice

**Fig. 3:**
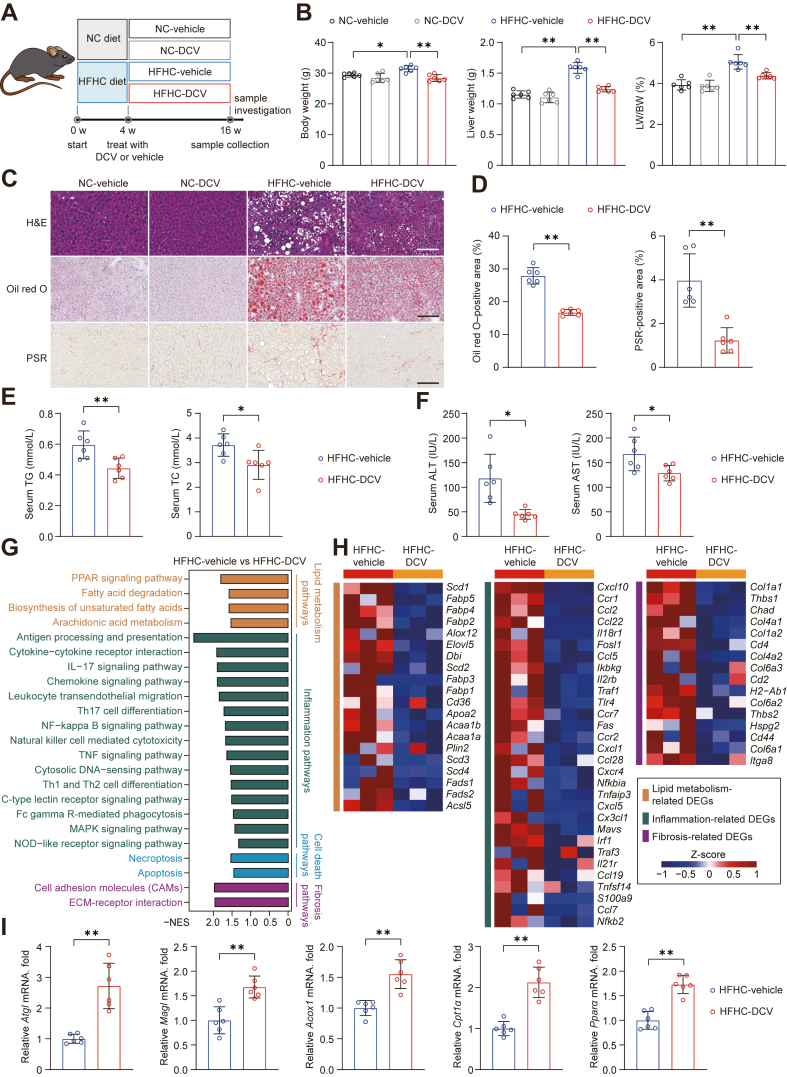
Daclatasvir treatment mitigates metabolic and inflammatory dysregulation in HFHC-induced MASH. A: Schematic illustration of the experimental workflow for the daclatasvir treatment strategy in a normal chow (NC), or a high-fat, high-cholesterol (HFHC) diet-induced MASH mouse model. B: Body weight, liver weight, and the ratio of liver weight to body weight (LW/BW) were recorded for NC or HFHC diet-fed mice after 12 weeks of treatment with either control solution or daclatasvir. C: Representative images of H&E, Oil Red O, and picrosirius red (PSR) staining of liver samples from vehicle or daclatasvir treated mice at 16 weeks of NC or HFHC diet administration. Scale bar, 200 μm. D: Quantitative results for Oil Red O and PSR staining shown in (C). E and F: Serum TG and TC concentrations (E), as well as enzyme activities of serum hepatic transaminases (F) in vehicle- or daclatasvir-treated mice after 16 weeks of HFHC feeding. G and H: KEGG enrichment analysis showed pathways (G) and heatmap illustrated the differential gene expression (H) related to lipid metabolism, inflammation and fibrosis, in livers from HFHC diet-induced MASH model treated with vehicle or daclatasvir. I: The mRNA expression of *Atgl*, *Magl*, *Acox1*, *Cpt1α*, and *Pparα* in liver tissues from HFHC diet-induced MASH mice following 12 weeks of daclatasvir treatment. For (B–F), and (I), n = 6 mice per group; for (G, H), n = 3 mice per group. Data are presented as mean ± SD; ∗*P* < 0.05, ∗∗*P* < 0.01; one-way ANOVA in (B), and Student’s *t* test in other statistics.

To explore the therapeutic effect and application potential of daclatasvir on MASH treatment, we subjected mice to either a NC or a HFHC diet for 4 weeks to induce the MASLD phenotype (22). Subsequently, mice were treated with either a vehicle (1% carboxymethylcellulose sodium, CMC-Na) or daclatasvir (10 mg/kg/day) for an additional 12 weeks with the dietary patterns maintained (Fig. 3A). Daclatasvir administration significantly attenuated body weight gain and reduced liver weight compared to vehicle control group after HFHC treatment (Fig. 3B). Histopathological analysis of liver tissue revealed that daclatasvir treatment decreased the content of hepatic lipid deposition and liver fibrosis compared to vehicle treatment (Fig. 3C, D). In line with improved liver features, the concentrations of serum TG and TC (Fig. 3E), as well as ALT and AST (Fig. 3F), were markedly reduced in the daclatasvir-treated group. Comprehensive transcriptome analysis of the livers from HFHC diet-fed mice indicated that daclatasvir effectively improved pathological pathways and gene panels related to lipid metabolism, inflammatory responses, cell death, and fibrosis (Fig. 3G, H). Furthermore, mice treated with daclatasvir also exhibited enhanced lipid catabolism, with higher mRNA expression levels of *Atgl*, *Magl*, *Acox1*, *Cpt1α*, and *Pparα* compared to those treated with vehicle after HFHC treatment for 16 weeks (Fig. 3I).

**Fig. 4:**
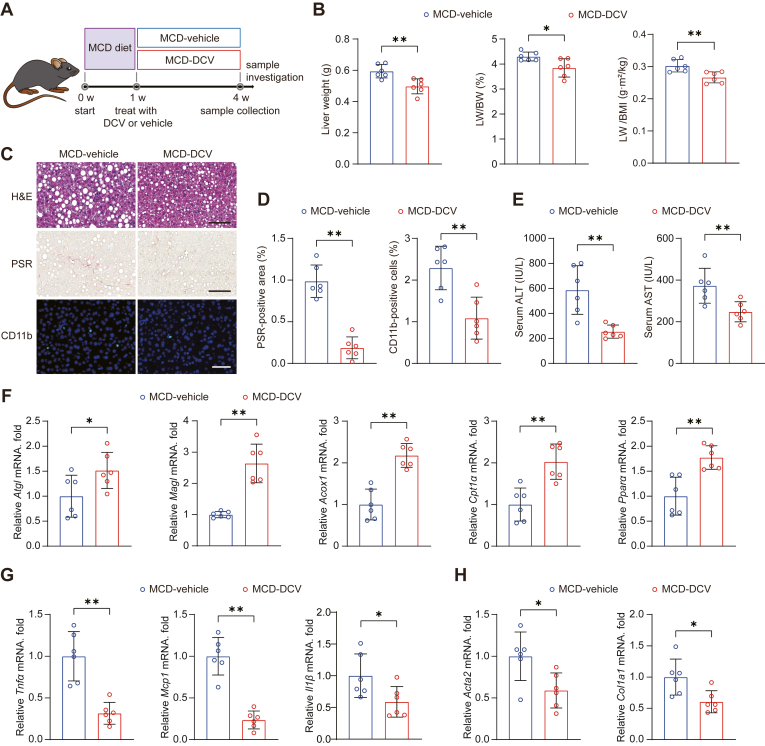
Daclatasvir treatment ameliorates liver damage and fibrosis in MCD diet-induced MASH mouse model. A: Schematic illustration of the experimental workflow for the daclatasvir treatment strategy in a methionine-choline deficient (MCD) diet-induced MASH mouse model. B: Liver weight, LW/BW, and LW/BMI were recorded for MCD diet-fed mice after 3 weeks of treatment with either vehicle or daclatasvir. C: Representative images of H&E, PSR, and CD11b staining of liver samples from vehicle or daclatasvir treated mice at 4 weeks of MCD diet administration. Scale bar, 200 μm for H&E and PSR; 100 μm for CD11b. D: Quantitative results for PSR and CD11b staining shown in (C). E: Enzyme activities of serum hepatic transaminases in MCD diet-fed mice after 3 weeks of treatment with either vehicle or daclatasvir. F–H: The mRNA expression of genes associated with lipid catabolism (F), inflammation (G), and fibrosis (H) in liver tissues from MCD diet-induced MASH mice following 3 weeks of daclatasvir or vehicle treatment. For (B–H), n = 6 mice per group. Data are presented as mean ± SD; ∗*P* < 0.05, ∗∗*P* < 0.01; Student’s *t* test for statistics.

Since inflammation and fibrosis represent major pathological characteristics responsible for the adverse outcomes of MASH (23), we further evaluated applicability of daclatasvir on methionine-choline deficiency (MCD) diet-induced MASH model with more advanced inflammatory response and fibrosis than HFHC model (24). In specific, mice were randomly divided into two groups after one week on the MCD diet. Both groups continued the MCD diet, with one group receiving CMC-Na and the other treated with daclatasvir at a dosage of 10 mg/kg (Fig. 4A). After four weeks of MCD administration, mice in the daclatasvir group exhibited significantly lower liver weight, LW/BW, and LW/BMI ratios compared to the vehicle group, indicating that daclatasvir effectively alleviates liver steatosis and liver damage (Fig. 4B). Histopathological analyses revealed substantial improvements in steatosis, fibrosis, and inflammation in the MCD-daclatasvir group (Fig. 4C, D), accompanied by significantly lower serum ALT and AST levels (Fig. 4E). The gene expression assay further validated that daclatasvir significantly promoted lipid hydrolysis and fatty acid oxidation while effectively reducing inflammation and fibrosis (Fig. 4F–H).

### Daclatasvir promotes the ubiquitination degradation of PLIN2

**Fig. 5:**
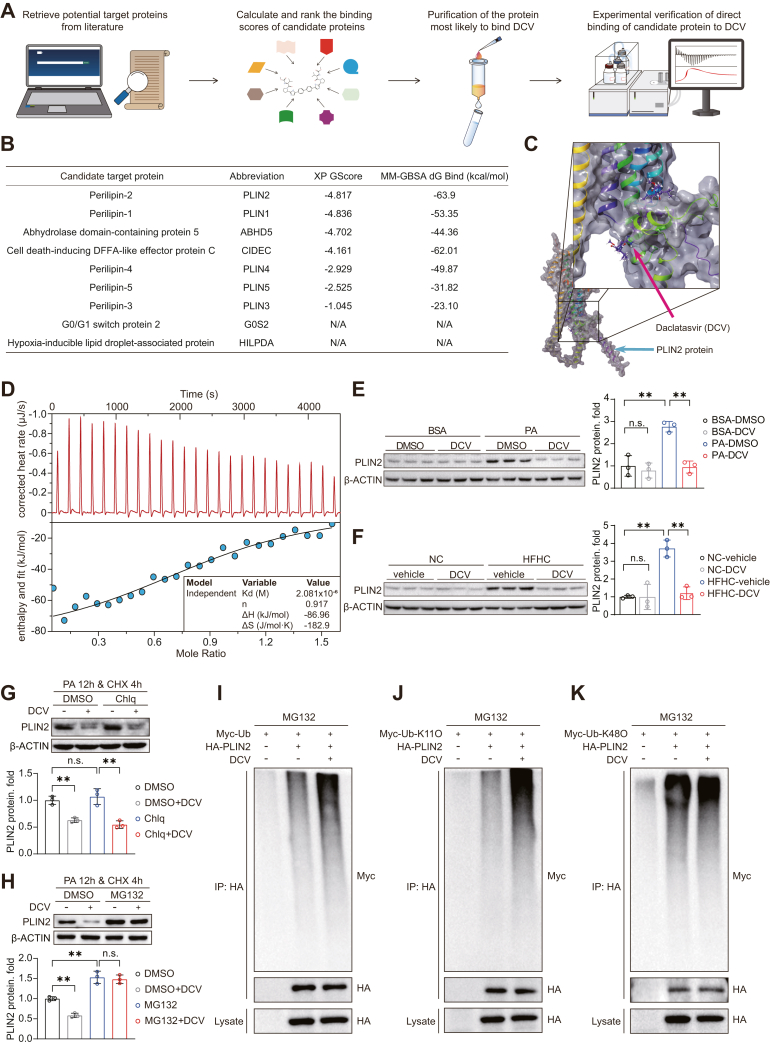
Daclatasvir targets PLIN2 for degradation via ubiquitination. A: The experimental workflow for screening potential target protein of daclatasvir. B: Table shows molecular docking analysis of nine candidate proteins with daclatasvir. The XP GScore reflects the binding affinity, and the MM-GBSA dG Bind reflects the binding free energy alterations upon complex formation. C: Molecular docking model demonstrating the interaction between daclatasvir and PLIN2 protein. The upper right box is a zoomed-in display of the lower left box. Red and blue arrows point to daclatasvir and PLIN2 proteins, respectively. D: Isothermal titration calorimetry analysis that illustrating the thermodynamic profiles of connection between daclatasvir and PLIN2 protein. E and F: Western blot analysis of PLIN2 protein levels in primary mouse hepatocytes treated with DMSO or daclatasvir and challenged with BSA or PA (0.5 mM) for 12 h (E), and those in liver tissues from NC or HFHC diet-fed mice treated with vehicle or daclatasvir (F). Quantitative data are shown on the right, using β-ACTIN as the loading control. G and H: PLIN2 protein levels in HuH-7 cells treated with DMSO or chloroquine (Chlq, 25 μM) (G), and those treated with DMSO or MG132 (10 μM) (H), following 12 h of PA (0.5 mM) stimulation and 4 h of cycloheximide (CHX, 100 μM) exposure. Quantitative data are shown on the bottom, using β-ACTIN as the loading control. I: Ubiquitination levels of PLIN2 in HEK 293T cells transfected with HA-tagged PLIN2 and Myc-tagged ubiquitin plasmids, treated with DMSO or daclatasvir (10 μM) in the presence of MG132 (10 μM). J and K: Ubiquitination levels of K11-linked PLIN2 (J) and K48-linked PLIN2 (K) in HEK 293T cells transfected with HA-tagged PLIN2 and Myc-tagged K11-only ubiquitin (Myc-Ub-K11O) (J) or Myc-tagged K48-only ubiquitin (Myc-Ub-K48O) (K) plasmids, treated with DMSO or daclatasvir (10 μM) in the presence of MG132 (10 μM). For (E–G), n = 3 independent biological replicates. Data are presented as mean ± SD; ∗*P* < 0.05, ∗∗*P* < 0.01, n.s., not significant; one-way ANOVA for statistics.

The primarily developed druggable target of daclatasvir is the HCV NS5A replication complex (13). Since the HCV infection is non-existent during MASH progression, we thus hypothesize that there might be other therapeutic target for the anti-MASH function of daclatasvir. To this end, we predicted several candidate proteins potentially binding to daclatasvir via a comprehensive literature search followed by molecular docking assay (Fig. 5A). Screening based on binding free energy and docking scores revealed that daclatasvir most stably binds to PLIN2 (Fig. 5B, C). Subsequent analysis using isothermal titration calorimetry (ITC) to measure heat changes during the binding of candidate proteins to daclatasvir clearly validated that PLIN2 exhibited direct interaction with daclatasvir in terms of binding strength and thermodynamic parameters, suggesting that PLIN2 might be function as a key target for daclatasvir. The binding stoichiometry (n) was found to be 0.917, indicating that approximately one daclatasvir molecule binds to one PLIN2 protein molecule (Fig. 5D).

Through in vivo and in vitro experiments, we found that daclatasvir effectively promotes PLIN2 degradation (Fig. 5E, F). To further investigate the pathway responsible for PLIN2 degradation, we blocked the influence of newly synthesized proteins using cycloheximide and then inhibited the lysosomal and proteasomal degradation pathways using chloroquine and MG132, respectively. The results showed a significant accumulation of PLIN2 following proteasome inhibition by MG132, instead of chloroquine, confirming that PLIN2 degradation predominantly occurs via the proteasome pathway (Fig. 5G, H).

Since ubiquitin-proteasome is key in regulating proteasomal degradation, we investigated whether daclatasvir promotes PLIN2 degradation by affecting its ubiquitination. Our ubiquitination assays confirmed that PLIN2 is mainly degraded via the proteasomal pathway. More importantly, daclatasvir markedly increased the ubiquitination level of PLIN2 to enhance its degradation (Fig. 5I). Furthermore, we found that daclatasvir treatment significantly increased the K11 chain ubiquitination of PLIN2, while K48 chain ubiquitination was unaffected (Fig. 5J, K).

### Daclatasvir enhances MARCH6 binding to PLIN2

**Fig. 6:**
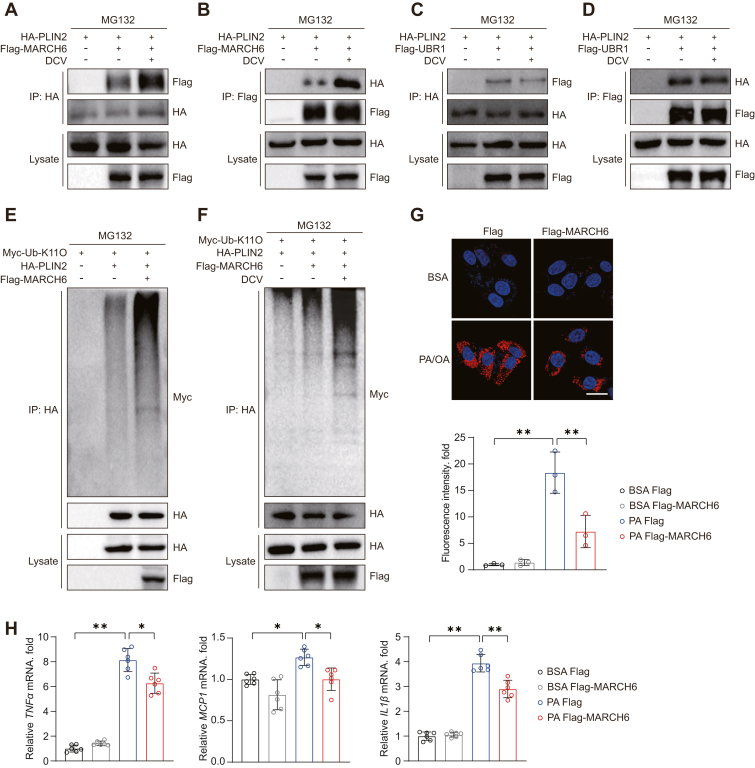
Daclatasvir enhances MARCH6-mediated PLIN2 ubiquitination to alleviate lipid accumulation and inflammation. A, B: coimmunoprecipitation (CoIP) assay of the interaction between MARCH6 and PLIN2 via immunoprecipitating HA-tagged PLIN2 (A) or Flag-tagged MARCH6 (B) in HEK 293T cells treated with DMSO or daclatasvir (10 μM) for 12 h following transfection with indicated plasmids. C and D: CoIP assay of the interaction between UBR1 and PLIN2 via immunoprecipitating HA-tagged PLIN2 (C) or Flag-tagged UBR1 (D) in HEK 293T cells treated with DMSO or daclatasvir (10 μM) for 12 h following transfection with the indicated plasmids. E: Ubiquitination levels of PLIN2 in HEK 293T cells transfected with HA-tagged PLIN2, Flag-tagged MARCH6, and Myc-tagged Ub-K11O, in the presence of MG132 (10 μM). F: Ubiquitination levels of PLIN2 in HEK 293T cells transfected with HA-tagged PLIN2, Flag-tagged MARCH6, and Myc-tagged Ub-K11O, treated with DMSO or daclatasvir (10 μM) in the presence of MG132 (10 μM). G: Representative Nile Red staining images of HuH-7 cells overexpressing MARCH6 or control plasmid and suffering 18 h of PA/OA (0.25 mM/0.5 mM) stimulation, along with quantitative analysis of lipid accumulation. Scale bar, 25 μm. H: The mRNA levels of *TNFα*, *MCP1*, and *IL1β* at 12 h post-PA (0.5 mM) stimulation in HuH-7 cells overexpressing MARCH6 or control plasmid. For (H), n = 6 independent biological replicates; for the others, n = 3 independent biological replicates. Data are presented as mean ± SD; ∗*P* < 0.05, ∗∗*P* < 0.01; one-way ANOVA for statistics.

PLIN2 has been reported to be subjected to ubiquitination mediated by MARCH6 or UBR1 (25, 26). To investigate how daclatasvir regulates PLIN2 ubiquitination, we explored the influence of daclatasvir on the binding of PLIN2 with its potential E3 ligases. Our coimmunoprecipitation experiments showed that daclatasvir significantly increased the interaction between PLIN2 and MARCH6 (Fig. 6A, B), while it did not affect the interaction between PLIN2 and UBR1 (Fig. 6C, D). Using the proteasome inhibitor MG132 in MARCH6-overexpressing cells, we observed that MARCH6 indeed increased the K11 ubiquitination of PLIN2 (Fig. 6E). Notably, daclatasvir treatment largely elevated K11 ubiquitination levels of PLIN2 mediated by MARCH6 (Fig. 6F). In line with the promoting effect of MARCH6 on PLIN2 ubiquitination and degradation, we found that overexpressing MARCH6 led to a significant reduction in lipid droplets in hepatocytes (Fig. 6G), indicating its key role in regulating lipid droplet metabolism. Additionally, qPCR results confirmed that MARCH6 effectively mitigates the inflammatory response in this model (Fig. 6H). These findings support that daclatasvir can function as a novel protein degradant by enhancing MARCH6-PLIN2 interaction and subsequent PLIN2 ubiquitinational degradation.

### The interaction with PLIN2 is essential for anti-MASH function of daclatasvir

**Fig. 7:**
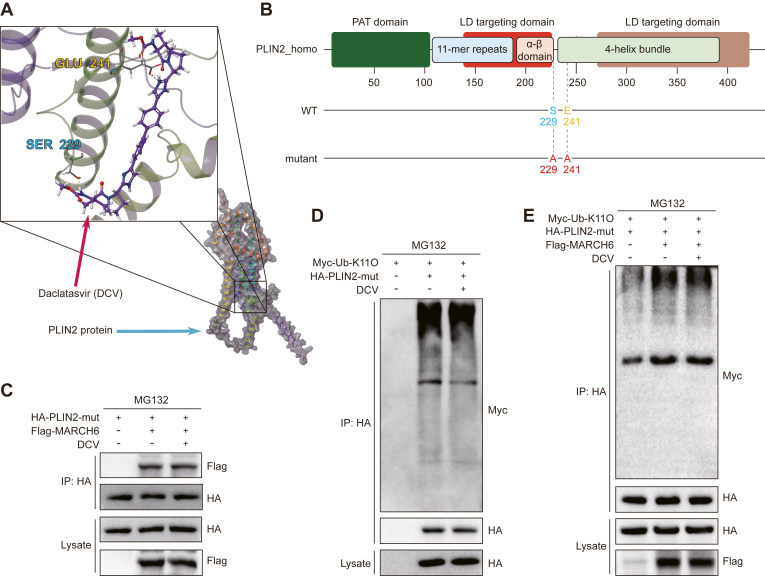
Daclatasvir enhances PLIN2 binding, ubiquitination, and interaction with MARCH6 via key residue mutations. A: Molecular docking model demonstrating the binding site (Ser229 and Glu241) of daclatasvir within the active pocket of PLIN2 protein. The upper left box is a zoomed-in display of the lower right box. Red and blue arrows point to daclatasvir and PLIN2 protein, respectively. B: Schematic representation of PLIN2 with mutations in the daclatasvir binding site (PLIN2-mut). PAT domain, Perilipin-ADRP-Tip47 domain; LD, lipid droplet; S, serine; E, glutamate; A, alanine. C: CoIP assay of the interaction between MARCH6 and PLIN2-mut by immunoprecipitating HA-tagged PLIN2-mut in HEK 293T cells treated with DMSO or daclatasvir (10 μM) for 12 h following transfection with the indicated plasmids. D: Ubiquitination levels of PLIN2-mut in HEK 293T cells transfected with HA-tagged PLIN2-mut, and Myc-tagged Ub-K11O, treated with DMSO or daclatasvir (10 μM) in the presence of MG132 (10 μM). E: Ubiquitination levels of PLIN2-mut in HEK 293T cells transfected with HA-tagged PLIN2-mut, Flag-tagged MARCH6, and Myc-tagged Ub-K11O, treated with DMSO or daclatasvir (10 μM) in the presence of MG132 (10 μM). All experiments were performed in three independent biological replicates.

In the present study, molecular docking analysis revealed that daclatasvir interacts with the active site of PLIN2, forming hydrogen bonds with Ser229 and Glu241 (Fig. 7A). To verify whether daclatasvir exerts its effects dependent on its binding to PLIN2, we constructed Ser229 and Glu241 mutant *PLIN2*-Mut plasmid (Fig. 7B). Notably, daclatasvir failed to enhance the interaction between PLIN2-Mut and MARCH6 (Fig. 7C) and to increase the K11 ubiquitination of PLIN2-Mut in the presence or absence of MARCH6 (Fig. 7D, E). These findings suggest that the regulatory action of daclatasvir on MASH is largely dependent on its binding to the Ser229 and Glu241 residues in PLIN2.

**Fig. 8:**
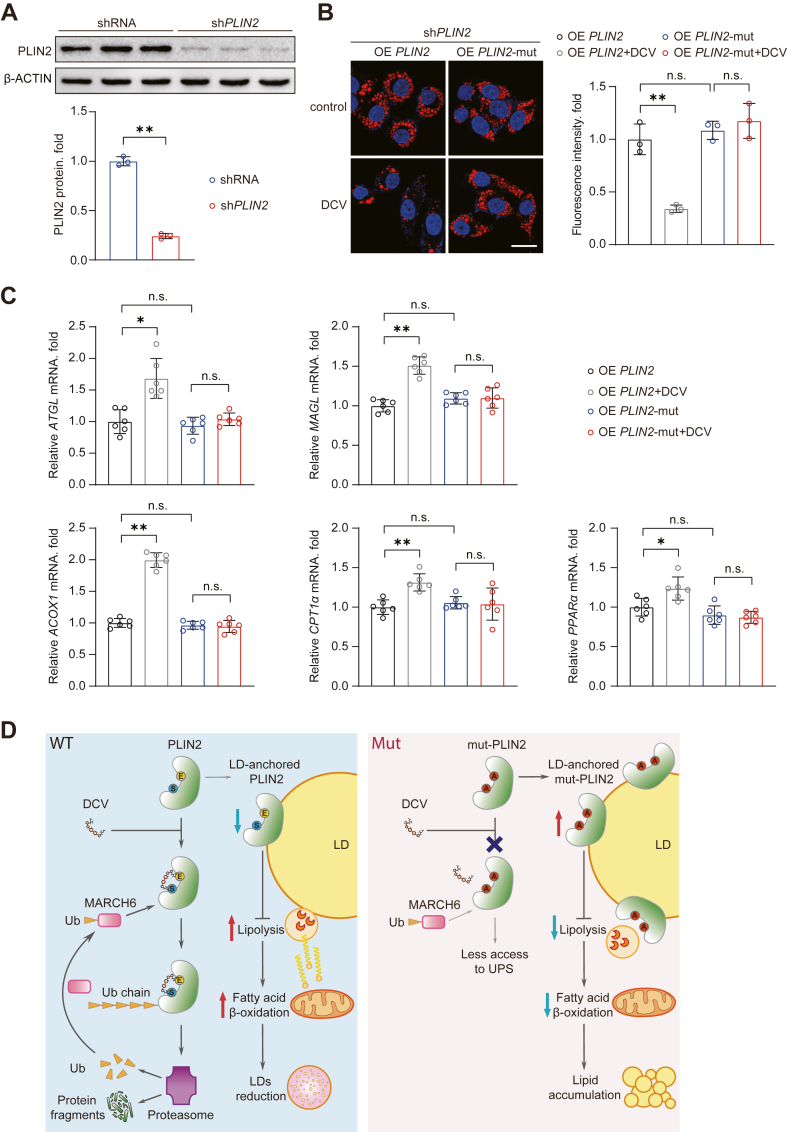
Daclatasvir reduces lipid accumulation by targeting PLIN2 and enhancing lipid metabolism pathways. A: PLIN2 protein levels in HuH-7 cells lentiviral infected with either control shRNA or sh*PLIN2*. Quantitative data are shown on the bottom, using β-ACTIN as the loading control. B: Representative Nile Red staining images of sh*PLIN2* HuH-7 cell lines with the reintroduction of *PLIN2* or *PLIN2*-mut plasmids, and treated with DMSO or daclatasvir (10 μM) for 12 h following 18 h of PA/OA (0.25 mM/0.5 mM) stimulation. Scale bar, 25 μm. C: The mRNA levels of lipolysis and fatty acid oxidation-related genes, including *ATGL*, *MAGL*, *ACOX1*, *CPT1α*, and *PPARα*, in sh*PLIN2* HuH-7 cell lines reintroduced with *PLIN2* or *PLIN2*-mut plasmids, treated with daclatasvir or DMSO for 12 h in presence of PA (0.5 mM) stimulation. D: Schematic representation illustrating that daclatasvir (DCV) binds to wild type (WT) PLIN2 in the cytoplasm, which facilitates MARCH6-mediated K11 ubiquitination of PLIN2. This process increases the access of PLIN2 to the ubiquitin (Ub)-proteasome system (UPS) and degradation, causes PLIN2 less bound to LD. It diminishes the protective effect of LD-anchored PLIN2, reducing the stability of LD and enhancing lipolysis, resulting in a reduction in lipid accumulation. Because of the non-interaction with daclatasvir, mutant (Mut) PLIN2 get less access to the UPS and anchors more into LD, thus keeping the stability of LD and inhibits lipolysis. For (A, B), n = 3 independent biological replicates; for (C), n = 6 independent biological replicates. Data are presented as mean ± SD; ∗*P* < 0.05, ∗∗*P* < 0.01, n.s., not significant; Student's *t* test in (A), and one-way ANOVA in (B, C).

To further illustrate whether PLIN2 is required for the function of daclatasvir, we carried out rescue experiments by reintroducing either wild-type PLIN2 or PLIN2 mutant plasmids in PLIN2 knockdown cell lines. Western blotting confirmed that PLIN2 expression was significantly reduced in the knockdown cells (Fig. 8A). We observed that PLIN2-mut showed comparable effect on lipid deposition as those induced by wild-type PLIN2 overexpression (Fig. 8B). Daclatasvir markedly and specifically reduced lipid accumulation in cells reintroduced with wild-type PLIN2 compared to controls, instead of in PLIN2 mutant plasmid transfected cells (Fig. 8B). In addition, qPCR analysis revealed that the transcript levels of genes associated with lipolysis and fatty acid β-oxidation were significantly elevated by daclatasvir in the wild-type PLIN2 overexpression group. However, this effect of daclatasvir was not reproduced in the PLIN2 mutant cell group (Fig. 8C). These findings suggest that daclatasvir specifically enhances lipolysis and fatty acid β-oxidation through functional PLIN2. Collectively, these results provide robust evidence that daclatasvir regulates lipid metabolism via a PLIN2-mediated mechanism.

## Discussion

This study addresses the urgent need for effective treatments for MASH by employing a drug repurposing strategy to identify novel therapeutic candidates. The drugs tested in our study were unbiasedly selected from the FDA-approved drug library based on their reported hepatoprotective effects as supported by previous literature and clinical studies (13, 27, 28, 29). As a result, most selected drugs with potential hepato-protective effect were antiviral drugs. Importantly, while these compounds are primarily classified as antiviral agents, emerging evidence suggests that some of them exert beneficial effects on liver health beyond their antiviral properties. We thus hypothesized that those selected drugs hold anti-MASH capacity by targeting lipid metabolic homeostasis. In this research, we systematically screened FDA-approved hepatoprotective drugs and identified daclatasvir as a promising candidate. Mechanistically, daclatasvir functions a promising protein degradant that directly binds to PLIN2 and promotes the MARCH6-mediated PLIN2 ubiquitinational degradation (Fig. 8D). Interrupting the binding between PLIN2 and daclatasvir fully abolished the protective effects of daclatasvir on MASH. These findings suggest that antiviral drugs may exert broader benefits on liver health beyond viral suppression, providing a novel therapeutic strategy to compensate the limitations of existing pharmacological treatments for MASLD.

Daclatasvir, originally developed for HCV therapy, inhibits viral replication by inducing a positional change of NS5A to allosterically interrupt its protein interactions within the replicase components and disrupt replicase assembly (13). Antiviral drugs, commonly used for treating viral hepatitis, have shown a potential to improve liver function lipid metabolism in hepatitis-associated fatty liver (30, 31), while the molecular mechanisms remain largely unknown. For example, rilpivirine, an anti-HIV drug, has demonstrated significant antifibrotic and anti-inflammatory effects in liver fibrosis models by selectively activating the STAT1 pathway within the JAK-STAT signaling cascade, leading to hepatic stellate cell (HSC) apoptosis. Furthermore, RPV promotes hepatocyte regeneration through paracrine-mediated activation of STAT3 (32). Similarly, alisporivir disrupts the viral life cycle by inhibiting host Cyclophilin A and modulating inflammatory responses, while miravirsen targets host miRNA-122 to suppress viral replication and improve lipid metabolism (33, 34). In the present study, we provide first evidence showing potent anti-MASH capacity of the anti-HCV drug daclatasvir. Daclatasvir effectively improved lipid metabolism, reduced inflammatory response, and alleviated liver fibrosis and dysfunction, paving a novel avenue for expanding clinical application of antiviral drugs.

In our pharmacological mechanism exploration, we clearly demonstrated that daclatasvir functions as a direct PLIN2 inhibitor. As an essential member of the perilipin protein family, PLIN2 plays a critical role in maintaining the structural integrity and stability of lipid droplets, dynamic lipid storage organelles responsible for metabolic homeostasis and lipotoxicity (35, 36). Recent studies reported that declining PLIN2 expression in the liver significantly improved high fat diet- and obesity-induced hepatic steatosis, inflammation, and fibrosis by enhancing ATGL-mediated lipolysis, autophagy, and fatty acid β-oxidation (37, 38). PLIN2 plays a key role in lipid droplet mobilization and metabolic reprogramming by regulating lipid droplet stability, lipid hydrolysis, and chaperone-mediated autophagic degradation. Specifically, PLIN2 forms a protective layer on the surface of lipid droplets to prevent the excessive release of fatty acids, while simultaneously relieving lipase inhibition to promote lipid breakdown. This dual function ensures the efficient supply of energy to the cell (39). However, excessive lipid hydrolysis can trigger lipidomic remodeling, particularly characterized by the reduction of cardiolipin and phosphatidylethanolamine levels. These changes disrupt mitochondrial function, impair fatty acid oxidation, and lead to altered gene expression and epigenetic modifications (36).The degradation of PLIN2 is mainly facilitated by ubiquitination. Among the components of the ubiquitination pathway, E3 ubiquitin ligases play a pivotal role by conferring substrate specificity, making them essential regulators of cellular processes and potential therapeutic targets for MASLD (40, 41, 42). Here, we for the first time identified the E3 ligase MARCH6 that robustly induces a previously unreported ubiquitinational type of PLIN2, the K11-type ubiquitination. In specific, MARCH6 directly binds to PLIN2 and promotes its K11-type ubiquitination and the subsequent protein degradation. Importantly, daclatasvir interacts with the Ser229 and Glu241 residues in PLIN2 and facilitates its interaction with MARCH6 to enhance PLIN2 K11-type ubiquitination and protein degradation. Mutation of these residues largely impairs the protective capacity of daclatasvir on PLIN2 degradation and MASLD progression.

In conclusion, this study reveals that daclatasvir possesses promising therapeutic capacity against MASLD by enhancing MARCH6-mediated degradation of PLIN2, a process dependent on Ser229 and Glu241 residues for PLIN2’s recognition by daclatasvir. These findings highlight potential application of antiviral drugs as an effective therapy for MASLD and related metabolic disorders.

## Data availability

All data are contained within the manuscript and supplementary data.

## Supplemental data

This article contains supplemental data.

## Conflict of interest

The authors declare that they have no conflicts of interest with the contents of this article.
