# Supplementary material for: Hepatoprotective drug screening identifies daclatasvir, a promising therapeutic candidate for MASLD by targeting PLIN2
Source: J Lipid Res. 2025 May 29;66(7):100835. doi: 10.1016/j.jlr.2025.100835 (PMC12268039; doi:10.1016/j.jlr.2025.100835)
Supplement: Supplementary Data 1 [file mmc1.docx]

**SUPPLEMENTAL INFORMATION:**

**Hepatoprotective drug screening identifies daclatasvir, a promising therapeutic candidate for MASLD by targeting PLIN2**

Rui Shu^1, ‡^, Song Tian^1,2, ‡^, Weiyi Qu^3, ‡^, Jinjie Yang^1,4, ‡^, Wei Shi^1^, Xinyan Li^1^, Toujun Zou^2^, Changjin Jiang^1^, Yuxuan Zhang^1^, Zifeng Yang^5^, Han Tian^5^, Hailong Yang^2^, Jiajun Fu^2^, Zhi-Gang She^2,5^, Hongliang Li^1,2,5,*^, Xiao-Jing Zhang^1,2,*^

^1^ Wuhan University TaiKang Medical School (School of Basic Medical Sciences), Wuhan, China

^2^ State Key Laboratory of New Targets Discovery and Drug Development for Major Diseases, Gannan Innovation and Translational Medicine Research Institute; School of Pharmacy, Gannan Medical University, Ganzhou, China

^3^ Department of Cardiology, Zhongnan Hospital of Wuhan University, Wuhan, China

^4^ Qujing Medical College Basic Medical Department, Qujing, China

^5^ Department of Cardiology, Renmin Hospital of Wuhan University, Wuhan, China

**Table S1. The gene sequence numbers.**

| **Gene** | **NM number** |
| --- | --- |
| *PLIN2* | NM_001122.4 |
| *MARCH6* | NM_001270660.2 |
| *UBR1* | NM_174916.3 |

**Table S2. Primer sequences for molecular cloning.**

| **Gene** | **Species** | **Forward primer (5’-3’)** | **Reverse primer (5’-3’)** |
| --- | --- | --- | --- |
| *PLIN2* | Human | TCGGGTTTAAACGGATCCATGGCATCCGTTGCAGTTGATC | GGGCCCTCTAGACTCGAGTTATTTGATGGCTTGGGAAGCA |
| *MARCH6* | Human | TCGGGTTTAAACGGATCCATGGACACCGCGGAGGAAG | GGGCCCTCTAGACTCGAGTTATTCTTGGGATGACTGTGGAGGTG |
| *UBR1* | Human | TCGGGTTTAAACGGATCCATGGCGGACGAGGAGGC | CCGCTCAAATACGGTCCTGAGAATGTAC |
| *PLIN2*-mut | Human | CTTAGCTGCTTTAACCCTGCTGAGAGCCTGCTGGTAGGCACGGGCGTGAA | CTTAGCTGCTTTAACCCTGCTGAGAGCCTGCTGGTAGGCACGGGCGTGAA |
| sh*PLIN2* | Human | CCGGCTTTAGATGACGTGATGGATTCTCGAGAATCCATCACGTCATCTAAAGTTTTTG | AATTCAAAAACTTTAGATGACGTGATGGATTCTCGAGAATCCATCACGTCATCTAAAG |

**Table S3. Primer sequences for RT-qPCR.**

| **Gene** | **Species** | **Forward primer (5’-3’)** | **Reverse primer (5’-3’)** |
| --- | --- | --- | --- |
| *Tnf-α* | Mouse | CATCTTCTCAAAATTCGAGTGACAA | TGGGAGTAGACAAGGTACAACCC |
| *Il-1β* | Mouse | CCGTGGACCTTCCAGGATGA | GGGAACGTCACACACCAGCA |
| *Mcp1* | Mouse | TACAAGAGGATCACCAGCAGC | ACCTTAGGGCAGATGCAGTT |
| *Col1a1* | Mouse | TGCTAACGTGGTTCGTGACCGT | ACATCTTGAGGTCGCGGCATGT |
| *Acta2* | Mouse | CCCAGACATCAGGGAGTAATGG | TCTATCGGATACTTCAGCGTCA |
| *Alcam* | Mouse | ATGGCATCTAAGGTGTCCCCT | CTGAGTTGACAGTGTACCATCC |
| *Atgl* | Mouse | TGGATGGCGGCATTTCAGACA | TGACGCGAAGCTCGTGGATGTT |
| *Magl* | Mouse | CGGACTTCCAAGTTTTTGTCAGA | GCAGCCACTAGGATGGAGATG |
| *Acox1* | Mouse | GTCTCCGTCATGAATCCCGA | TGCGATGCCAAATTCCCTCA |
| *Cpt1α* | Mouse | AGGACCCTGAGGCATCTATT | ATGACCTCCTGGCATTCTCC |
| *Pparα* | Mouse | TATTCGGCTGAAGCTGGTGTAC | CTGGCATTTGTTCCGGTTCT |
| *Actb* | Mouse | GTGACGTTGACATCCGTAAAGA | GCCGGACTCATCGTACTCC |
| *TNF-α* | Human | TGGCGTGGAGCTGAGAGATA | TGATGGCAGAGAGGAGGTTG |
| *IL-1β* | Human | ATGATGGCTTATTACAGTGGCAA | GTCGGAGATTCGTAGCTGGA |
| *MCP1* | Human | ATAGCAGCCACCTTCATTCCC | CAGCTTCTTTGGGACACTTGC |
| *ATGL* | Human | CTCCACCAACATCCACGAG | CCCTGCTTGCACATCTCTC |
| *MAGL* | Human | AATGCAGACGGACAGTACCTC | GAGCCAGCTCTTCATAGCGG |
| *ACOX1* | Human | TGGCCGCTATGATGGGAATG | CAGTGACTTCAGGTGCTTGT |
| *CPT1α* | Human | TCCAGTTGGCTTATCGTGGTG | CTAACGAGGGGTCGATCTTGG |
| *PPARα* | Human | ATGGTGGACACGGAAAGCC | CGATGGATTGCGAAATCTCTTGG |
| *ACTB* | Human | CATGTACGTTGCTATCCAGGC | CTCCTTAATGTCACGCACGAT |

**Table S4. Antibodies used in the experiment.**

| **Antibodies** | **Supplier** | **Catalog Number** |
| --- | --- | --- |
| Rabbit anti-Flag | Biolight | TP00975GeA20 |
| Mouse anti-Flag | Biolight | TM00975GeM20-F1F |
| Rabbit anti-HA | Biolight | TP00973GeA10 |
| Mouse anti-HA | Biolight | TM00973GeM20-C6F |
| Rabbit anti-β-ACTIN | Cell Signaling Technology | 4970T |
| Mouse anti-β-ACTIN | Cell Signaling Technology | 3700T |
| Rabbit anti-Myc | Biolight | TP00092GeA10 |
| Mouse anti-Myc | Biolight | TM00092GeM10-G3F |
| Rabbit anti-PLIN2 | Proteintech | 15294-1-AP |
