# Supplementary material for: Hepatoprotective drug screening identifies daclatasvir, a promising therapeutic candidate for MASLD by targeting PLIN2
Source: J Lipid Res. 2025 May 29;66(7):100835. doi: 10.1016/j.jlr.2025.100835 (PMC12268039; doi:10.1016/j.jlr.2025.100835)
Supplement: Supplementary Data 2 [file mmc2.pdf]

<u>Form for Submitting Manuscript</u>

Statement of Authorship and Disclosure of Potential Conflict of Interest Form

In the following manuscript, we declare we have made an important scientific contribution to this study, and we are familiar with the primary data; all co-authors have read the entire manuscript and take responsibility for its content.

Title of article:

<u>Hepatoprotective drug screening identifies daclatasvir a promising therapeutic candidate for MASLD by targeting PLIN2</u>

First Author: Rui Shu, Song Tian, Weiyi Qu, Jinjie Yang,

Corresponding Author: Hongliang Li, Xiao-Jing Zhang

List of all authors and include signatures of each

	Author’s Printed Name	Signature	Date signed
1	Rui Shu	Rui Shu	2025.5.10
2	Song Tian	Song Tian	2025.5.10
3	Weiyi Qu	Weiyi Qu	2025.5.10
4	Jinjie Yang	Yang JinJie	2025.5.10
5	Wei Shi	Wei Shi	2025.5.10
6	Xinyan Li	Xinyan Li	2025.5.10
7	Toujun Zou	Toujun Zou	2025.5.10
8	Changjin Jiang	Changjin Jiang	2025.5.10
9	Yuxuan Zhang	Yuxuan Zhang	2025.5.10
10	Zifeng Yang	Zifeng Yang	2025.5.10
11	Han Tian	Tian Han	2025.5.10
12	Hailong Yang	Hailong Yang	2025.5.10
13	Jiajun Fu	Jiajun Fu	2025.5.10
14	Zhi-Gang She	[illegible]	2025.5.10
15	Hongliang Li	[illegible]	2025.5.10
16	Xiao-Jing Zhang	Xiaojing Zhang	2025.5.10

[1] Wuhan University TaiKang Medical School (School of Basic Medical Sciences), Wuhan, China

[2] State Key Laboratory of New Targets Discovery and Drug Development for Major Diseases, Gannan Innovation and Translational Medicine Research Institute; School of Pharmacy, Gannan Medical University, Ganzhou, China

[3] Department of Cardiology, Zhongnan Hospital of Wuhan University, Wuhan, China

[4] Qujing Medical College Basic Medical Department, Qujing, China

[5] Department of Cardiology, Renmin Hospital of Wuhan University, Wuhan, China

		Email
1	Rui Shu	shurui@whu.edu.cn
2	Song Tian	tiansong007@whu.edu.cn
3	Weiyi Qu	whuquweiyi@163.com
4	Jinjie Yang	yangjinjie2017@163.com
5	Wei Shi	9202401028@fjmu.edu.cn
6	Xinyan Li	lixinyan@whu.edu.cn
7	Toujun Zou	zoutj@gimi.ac.cn
8	Changjin Jiang	525144191@qq.com
9	Yuxuan Zhang	zyx1379896387@163.com
10	Zifeng Yang	2021203020006@whu.edu.cn
11	Han Tian	tianhan2022@163.com
12	Hailong Yang	yanghl@gmu.edu.cn
13	Jiajun Fu	fujiajun@gmu.edu.cn
14	Zhi-Gang She	zgshe@whu.edu.cn
15	Hongliang Li	lihl@whu.edu.cn
16	Xiao-Jing Zhang	zhangxjing@whu.edu.cn
